# Age of Speech Onset in Autism Relates to Structural Connectivity in the Language Network

**DOI:** 10.1093/texcom/tgaa077

**Published:** 2020-10-23

**Authors:** Elise B Barbeau, Denise Klein, Isabelle Soulières, Michael Petrides, Boris Bernhardt, Laurent Mottron

**Affiliations:** Cognitive Neuroscience Unit, Montreal Neurological Institute, McGill University, Montreal, QC H3A 2B4, Canada; Neurology and Neurosurgery Department, McGill University, Montreal, QC H3A 2B4, Canada; Center for Research on Brain, Language and Music (CRBLM), Montreal, QC H3G 2A8, Canada; Cognitive Neuroscience Unit, Montreal Neurological Institute, McGill University, Montreal, QC H3A 2B4, Canada; Neurology and Neurosurgery Department, McGill University, Montreal, QC H3A 2B4, Canada; Center for Research on Brain, Language and Music (CRBLM), Montreal, QC H3G 2A8, Canada; Department of Psychology, Université du Québec à Montreal, Montreal, QC H2X 3P2, Canada; Cognitive Neuroscience Unit, Montreal Neurological Institute, McGill University, Montreal, QC H3A 2B4, Canada; Neurology and Neurosurgery Department, McGill University, Montreal, QC H3A 2B4, Canada; Department of Psychology, McGill University, Montreal, QC H3A 1G1, Canada; Neurology and Neurosurgery Department, McGill University, Montreal, QC H3A 2B4, Canada; Département de Psychiatrie et d’addictologie, de l'Université de Montréal, Montréal, QC H3T 1J4, Canada; Centre de recherche du CIUSSS-NIM, Montreal, QC H1E 1A4, Canada

**Keywords:** arcuate fasciculus, autism, diffusion imaging tractography, language, speech onset delays

## Abstract

Speech onset delays (SOD) and language atypicalities are central aspects of the autism spectrum (AS), despite not being included in the categorical diagnosis of AS. Previous studies separating participants according to speech onset history have shown distinct patterns of brain organization and activation in perceptual tasks. One major white matter tract, the arcuate fasciculus (AF), connects the posterior temporal and left frontal language regions. Here, we used anatomical brain imaging to investigate the properties of the AF in adolescent and adult autistic individuals with typical levels of intelligence who differed by age of speech onset. The left AF of the AS group showed a significantly smaller volume than that of the nonautistic group. Such a reduction in volume was only present in the younger group. This result was driven by the autistic group without SOD (SOD−), despite their typical age of speech onset. The autistic group with SOD (SOD+) showed a more typical AF as adults relative to matched controls. This suggests that, along with multiple studies in AS-SOD+ individuals, atypical brain reorganization is observable in the 2 major AS subgroups and that such reorganization applies mostly to the language regions in SOD− and perceptual regions in SOD+ individuals.

## Introduction

An altered developmental trajectory of brain connectivity is a commonly suspected mechanism to account for the cognitive and behavioral manifestations of individuals in the autism spectrum (AS) (see Muller et al. 2011; Vissers et al. 2012 for reviews; Uddin et al. 2013; Di Martino et al. 2014; Picci et al. 2016; Hong et al. 2019a). Altered brain connectivity is most frequently reported between frontoposterior functional regions (Kana et al. 2006; Just et al. 2012). Atypical left inferior frontal gyrus (IFG) and left superior temporal gyrus activation during a sentence comprehension task (Just et al. 2004; Kana et al. 2006) suggests that reorganization of this network plays a role in autistic language manifestations (see also Just et al. 2007; Schipul et al. 2011; Tyszka et al. 2014).

The speech phenotype in AS individuals is extremely variable. Although it does not contribute to the autism diagnosis according to DSM-5 criteria, speech onset delay (SOD) followed by language atypicalities remains one of the main characteristics of the most stringent autism phenotype (Wodka et al. 2013). However, certain AS individuals, broadly overlapping with the previous Asperger syndrome category, display opposite early speech milestones and high-level syntactic abilities. The presence (SOD+) or absence (SOD−) of SOD has a lifelong impact on cognitive function (Barbeau et al. 2013), the nature of peaks of abilities or outstanding skills in a specific domain (e.g., visuospatial or auditory; Bonnel et al. 2010), the intelligence subtests profile (e.g., Block Design or Similarities scores above the individual’s Full-scale IQ score; Soulieres et al. 2011), motor abilities (Barbeau et al. 2015a), and reported domains of interest (Chiodo et al. 2017). AS-SOD+ individuals show enhanced activation in perceptual expertise areas, even in higher-level cognitive tasks that are not purely perceptual and that rely on frontal areas in typically developing individuals. Conversely, AS-SOD− individuals show more widespread activation of language areas than AS-SOD+ individuals when listening to speech-like sounds (Samson et al. 2015). Structurally, specific differences in gyrification in the temporal and occipital brain areas between groups of AS-SOD+ and SOD− individuals have been reported (Duret et al. 2018).

In light of the above-mentioned evidences and because of the importance of the lateral frontal cortex and posterior temporal areas in language processing and learning (Ardila et al. 2016), it is unquestionably relevant to investigate specifically the connectivity between these regions in relation to language development in AS.

The white matter fibers that connect frontal and temporal language areas form the arcuate fasciculus (AF). The AF is the main white matter tract that connects posterior temporal language processing regions with the inferior frontal cortex. This dorsal language network is refined from childhood to adulthood in association with language processing abilities (Brauer et al. 2011): Children use a more extended network to process language, which becomes more confined in adults (Brauer et al. 2011). In typical development, the structural properties of the AF have been linked with word acquisition (Lopez-Barroso et al. 2013), vocabulary growth (Su et al. 2018), and other linguistic abilities (Salvan et al. 2017). Lesions to the fibers of the AF often result in conduction aphasia, leading to impairments in the repetition of speech, phonological paraphasia, and impaired speech monitoring and learning (Damasio and Damasio 1980; Bernal and Ardila 2009).

Consistent with the persistent finding of differences in structure–function relationship in the autistic brain, some evidence suggests that language impairments (LI) in AS may not be associated with the same neuroanatomical substrate as LIs in non-AS individuals. For example, Verly et al. (2014) used resting-state functional connectivity to investigate the language network in relation to language performance and impairments in AS-LI adolescents (with language delay and/or LI). In that sample, preserved intrahemispheric functional connectivity was observed between the frontal and temporal language areas. Lower language performance in those individuals was related with reduced interhemispheric connectivity as well as reduced connectivity between the cerebellum and the language areas. Verhoeven et al. (2012) compared the properties of dorsal language tracts (including the superior longitudinal and arcuate fasciculi) in AS-LI versus specific language impairments (SLI) adolescents. Compared with age-matched control groups, the AS-LI group did not differ, but the SLI group had significantly reduced fractional anisotropy (FA) in the dorsal pathway that was related to lower language subtest scores. In the AS-LI group, there was no correlation between any language score and the properties of the tracts. Another study investigated structure-function relationships in the language network in AS individuals comparing the AF white matter microstructural properties, with a magnetoencephalography measure associated with passive sound processing and which is a precursor to language processing (the auditory mismatch field). Delayed auditory mismatch field was associated with greater LI and AF microstructural properties in typical but not in an AS subgroup (Berman et al. 2016).

Looking more closely into the literature on structural differences of the AF in AS brings out inconsistencies (Travers et al. 2012 for a review). One factor that may explain this variability is a regression to the mean in larger samples, which when divided into subsamples, leads to contradictory results (Lombardo et al. 2019; Rodgaard et al. 2019). The age of participants is also an important factor in the large variability in neuroimaging results in autism. For example, the pattern of functional over- or underconnectivity found in autism depends on the age group studied and even switches to the opposite direction from childhood to adolescence, followed by normalization into adulthood (when differences in connectivity are no longer observed) (Nomi and Uddin 2015). Liu et al. (2019) examined structural connectivity of the dorsal language tracts in 6-week-old infants at-risk for autism, and although they did find between-group differences in other white matter tracts (superior longitudinal fasciculus [SLF]), no group differences were observed for the AF. In toddlers, FA in the AF has been found to be bilaterally reduced in an autistic sample with a history of language regression (Zhang et al. 2018). A reduced number of streamlines and increased diffusivity in the long segment of the left AF connecting temporal and frontal areas was also reported in AS adults, but reductions were not associated with degrees of communication impairments scores and the study did not specify whether participants had experienced SOD or not (Catani et al. 2016). Reduced AF volume was observed in a group of adults largely composed of Asperger individuals (Moseley et al. 2016). Overall, although atypical AF properties have been described in some AS samples, associations between language acquisition history and AF structural connectivity remain unclear.

This study aims to investigate specifically whether having or not delayed acquisition of speech in AS is related to the properties of the AF, a white matter tract involved in language development in typical populations. We used diffusion imaging tractography of the AF in autistic individuals whose age of onset of first words and current verbal ability level varied, taking their age at testing into account. In order to investigate how eventual structural differences in the white matter fibers relate to the properties of the cortical areas they connect to, we also looked at the gray matter volume of target frontal and temporal areas. We hypothesized that the properties of the left AF would differ in AS individuals according to whether they had SOD or not. Moreover, because of the phenotypic convergence of AS-SOD+ and AS-SOD− individuals in adulthood, we expected that the differences in structural connectivity would be more pronounced in younger than older individuals.

## Materials and Methods

### Participants

This study included 28 typically developing individuals and 34 AS individuals (Table 1), all recruited from the research database of the Autism Cognitive Neurosciences lab at Rivière-des-Prairies Hospital (University of Montreal, Montreal, Canada). All participants were right-handed (as assessed with the Edinburg Handedness Inventory; Oldfield 1971) and male, and the 2 groups were matched for age (14–35 years) and intellectual functioning (Wechsler Performance IQ (PIQ)). Within the AS sample, 18 had SOD (SOD+), defined by being >24 months old at the onset of their first words and/or >33 months old for their first 2-word sentence, and 16 developed speech at a typical age (SOD−). All AS participants were diagnosed by a multidisciplinary team of experienced clinicians and met DSM-IV criteria (American Psychological Association 1994) for autism. The evaluation included the Autism Diagnostic Interview-Revised (ADI-R; Lord et al. 1994) and/or Autism Diagnostic Observation Schedule (ADOS-G; Lord et al. 2000), clinical assessment, and psychometric testing, including the Raven Progressive Matrices and Weschler IQ test. Participants were allocated to SOD + or − according to ADI questions on speech onset history at time of diagnosis. Typically developing participants were recruited from the same community but did not have personal and/or familial neurological, psychiatric, or medical conditions known to affect brain function. Exclusion criteria for all participants were the current use of psychoactive or vasoactive medication and the use of drugs or alcohol (exceeding 2 drinks a day). The sample included participants from 2 protocols from our laboratory (Soulieres et al. 2009; Barbeau et al. 2015b). All participants or their parents gave written informed consent and received compensation for their time in accordance with Regroupement Neuroimagerie Quebec ERB approved protocols #06-07-018 or #08-09-003.

**Table 1:** Participants characteristics

	TYP	AS			*p*-value	
		Total	SOD+	SOD−	TYP vs. AS	SOD+ vs. SOD−
*n*	28	34	18	16		
Age (SD)	22.8 (5.3)	20.1 (5.5)	20.7 (6.4)	19.5 (4.6)	0.062	0.548
Raven score	49.3	48.8	48.0	49.7	0.785	0.444
Performance IQ	105.1	104.9	105.6	104.1	0.986	0.776
Full-scale IQ	107.7	101.3	98.8	104.1	0.398	0.321
Verbal IQ	108.8	99.4	93.9	105.5	0.009	0.121
Similarities	11.6	10.4	9.8	11.2	0.038	0.090
Comprehension	10.8	7.6	6.2	9.6	<0.001	0.010
Block Design	12.2	12.2	13.2	10.7	0.284	0.035
Age first words	—	25.3	35.4	14.5	—	<0.001
Age first sentences	—	34.1	45.1	23.0	—	<0.001

### Magnetic Resonance Imaging (MRI) Data Collection

MRI data were acquired on a Siemens Tim Trio 3 T scanner at the Unité de Neuroimagerie Fonctionnelle (University of Montreal) as part of 2 separate experiments. The first experiment included 20 typical and 18 AS (5 SOD−, 13 SOD+) participants (Barbeau et al. 2015b) and the imaging session, using a 32-channel head coil, included a 3D-ME-MPRAGE *T*_1_-weighted anatomical scan (176 slices, 1 mm^3^ voxels, repetition time [TR] = 2530 ms, echo time [TE] = 1.64/3.5/5.36/7.22 ms) and a 20:32-min 2D echo-planar imaging diffusion-weighted scan (128 directions, *b* values = 0 and 700 s/mm^2^, TR = 8740 ms, TE = 83 ms, 70 axial slices, FoV 256 mm, 2-mm interleaved slices, voxel size 2 mm^3^, PAT mode: GRAPPA, PAT factor: 2). The second experiment included 8 typical and 16 AS (11 SOD−, 5 SOD+) participants (Soulieres et al. 2009) and the imaging session using an 8-channel head coil included a 3D-MPRAGE *T*_1_-weighted anatomical scan (176 slices, TR = 2530 ms, TE = 3.48 ms, 1 mm^3^) and a 15:02-min 2D echo-planar imaging diffusion-weighted scan (60 directions, *b* values = 0 and 700 s/mm^2^, TR = 12 700 ms, TE = 100 ms, 75 axial slices, FoV 256 mm, 2-mm interleaved slices, voxel size 2 mm^3^, no PAT mode).

### MRI Data Analysis

#### Diffusion Imaging Preprocessing and Tract Dissection

**Figure 1:**
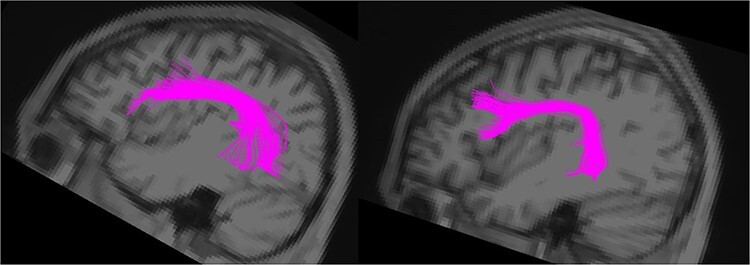
Final arcuate fasciculus (AF) dissection in 2 participants.

Diffusion imaging processing was based on FSL tools. In brief, FLIRT was used for coregistration of *T*_1_-weighted anatomical and diffusion-weighted images (DWIs) in native space, followed by eddy-current correction and brain extraction. Voxel-wise diffusion tensor estimation and whole-brain deterministic tractography (max threshold: 35^o^, min FA: 0.2) were performed using Diffusion Toolkit and Trackvis software. The main tract of interest, the left AF, was dissected for every participant using specific region of interest (ROI) placements, as described in Barbeau et al. 2020, to include the fibers connecting specifically the lateral frontal areas with the posterior part of the temporal lobe (see Fig. 1 for final result). The right arcuate and left superior longitudinal fasciculi branch III (connecting the frontal areas with the supramarginal gyrus of the inferior parietal lobe, ROI placement methods also described in Barbeau et al. 2020) were also dissected as control tracts. The volume, number of streamlines, and FA were extracted for each dissected tract and participant. One AS participant, whose AF values were 2.5 standard deviations (SDs) outside the average of the group, was removed.

### Plan of the Analyses

We first used diffusion tractography of the AF to investigate anatomical connectivity between the left posterior temporal and left inferior frontal language areas in individuals on the AS versus that of typically (TYP) developing individuals. Then, the AS group was separated into AS-SOD+ and AS-SOD− to investigate whether the 2 subtypes differ in the properties of the AF. We also investigated the effect of age (at testing) and assessed whether our results differed in adolescents/young adults versus older adults. We finally examined how these measures relate to behavioral, language, and communication measures to help further interpret group differences in connectivity.

### Additional Sensitivity Analyses

#### Voxel-Based Morphometry (VBM)

A VBM analysis (SPM8 VBM-DARTEL; Ashburner 2010) was also performed to confirm that regional group differences observed in the patterns of brain connectivity were not related to gray matter volume in the target brain areas connected by the AF. The *T*_1_-weighted images were first visually inspected for artifacts and then segmented into gray matter, white matter, and cerebrospinal fluid. The resulting individual gray matter images were used in the DARTEL pipeline and the resulting template files were smoothed (10-mm FWHM Gaussian kernel), spatially normalized, and scaled to MNI152 space. A one-way ANOVA was performed to examine group differences in whole-brain gray matter volume. ROI analyses were also performed to examine whether differences in cortical volume could be found within the connected regions. The ROIs of the IFG (BA 44 and 45) and the temporal ROIs (Brodmann areas (BA) 41–42 and BA 22) were created using the WFU PickAtlas (Maldjian et al. 2003). A corrected family-wise error critical threshold of *P*_FWE_ < 0.05 was used across the entire intracranial volume. The gray matter density within each ROI was also extracted for each participant for use in correlation analyses with anatomical connectivity measures and cognitive scores.

#### Motion

Using FSL’s Mcflirt, we computed the motion parameters for the DWI scans using the first volume as reference to ensure that differences in motion between groups did not account for the results observed. Maximum rotation and translation values were extracted from the Mcflirt.par files for each participant and independent-sample *t*-tests did not reveal any difference in the amount of motion (Table 2).

**Table 2:** Average maximal and minimal rotation (degrees) and translation (mm) motion values for each group for the DWI scans. *P*-values from the independent *t*-test are presented

			TYP	AS	*P*
DWI	rot	Max	1.23	0.96	0.308
		Min	−1.25	−1.21	0.867
	trans	Max	1.77	1.60	0.448
		Min	−0.62	−0.69	0.222

#### Controlling for Experiment

Since our sample included participants from 2 different imaging protocols, we compared all participants from protocol 1 with all those from protocol 2, each including AS and TYP individuals, to ensure that the differences found were not due to technical differences in the acquisition between experiments.

## Results

### Tractography

#### Group-Dependent Difference in the AF (AS vs. TYP)

**Figure 2:**
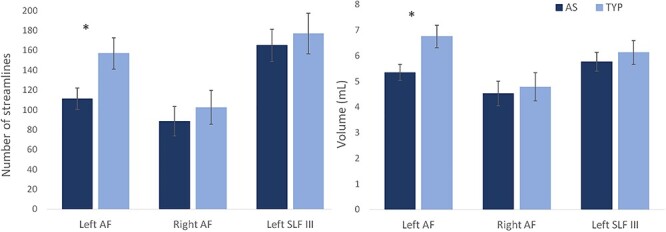
Average values for the autism spectrum (AS) and typically developing (TYP) groups for the left and right arcuate fasciculus (AF) and the left third branch of the superior longitudinal fasciculus (SLF III). The left AF number of streamlines and volume are significantly smaller in the AS group compared with the TYP group.

Independent sample *t-*tests showed a smaller number of streamlines of the left AF in AS compared with the TYP group (AS: *M* = 111.15, SD = 62.8; TYP: *M* = 156.9, SD = 83.2; *t* = −2.465, *P* = 0.017), as well as a smaller volume (AS: *M* = 5.34 mL, SD = 1.8; TYP: *M* = 6.75 mL, SD = 2.34; *t* = −2.662, *P* = 0.010; Fig. 2).

Even though we were unable to dissect the right AF for certain (25%) participants, which is common in deterministic tractography investigations of the AF (Catani et al. 2007; Eluvathingal et al. 2007; Lebel and Beaulieu 2009), we still examined the group-level difference in the right AF to determine whether the results were specific to the left hemisphere. A second control tract was used in the left hemisphere, the SLF, which runs along the AF in the frontal lobe but terminates in the inferior parietal lobe. There were no differences in the right AF and no differences in the left SLF between groups (Fig. 2). There were also no group-level differences in terms of microstructural properties for any of the tracts. The values for each tract and group and *P* values from *t*-tests are presented in Supplementary Table 2.

#### Effect of SOD

**Figure 3:**
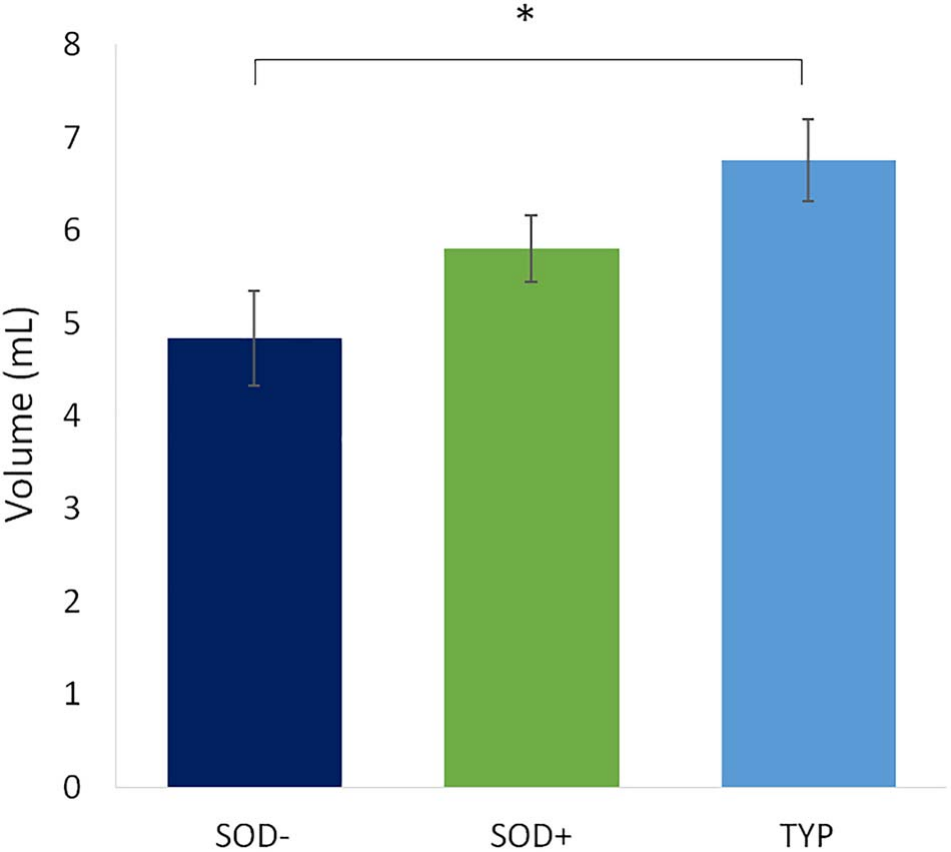
Group difference in AF Volume when the AS group is separated into SOD+ and SOD−. The SOD− group has significantly smaller AF volume than the TYP group.

We separated the AS group into SOD+ and SOD− subgroups to test whether early language experience has an effect on the development of the AF and to determine whether the reduction in the number of streamlines and volume of the AF was common to all AS subtypes that experience a different speech development history. An ANOVA revealed a significant group effect for the number of streamlines (*F* = 3.273, *P* = 0.045) and volume (*F* = 4.533, *P* = 0.015) of the AF. Planned contrasts showed that the SOD− group drove the AF reduction within the AS group: nb streamlines SOD− < TYP (Bonferroni *P* = 0.056, Dunnet’s *t*-test: *P* = 0.018, Cohen’s *d* = 0.67), SOD+ ≈ TYP (Dunnet’s *t*-test: *P* = 0.088, Cohen’s *d* = 0.45); volume SOD− < TYP (Bonferroni *P* = 0.013, Dunnet’s *t*-test: *P* = 0.004, Cohen’s *d* = 0.83), SOD+ ≈ TYP (Dunnet’s *t*-test *P* = 0.111, Cohen’s *d* = 0.54; Fig. 3). There were no group differences for any other metrics such as FA (*F* = 0.469, *P* = 0.628) or mean diffusivity (MD: *F* = 0.023, *P* = 0.978).

#### Effect of MRI Scanning Protocol

An independent sample *t*-test, comparing all the participants (AS and TYP) who did protocol 1 with all participants who did protocol 2, did not reveal any group differences in FA (*t* = −245, *P* = 0.807), the number of streamlines (*t* = −0.799, *P* = 0.427), or volume (*t* = −1.147, *P* = 0.256) of the AF. The same comparison was made for each group and subgroup separately and did not reveal any group differences (see Supplementary Table 1 for results). An analysis of covariance (ANCOVA) with scanning protocol as a covariate increases the significance of the group differences both for number of streamlines (*F* = 4.383, *P* = 0.017) and volume (*F* = 6.957, *P* = 0.002). There is a tendency for tract values to be lower for protocol 1 than protocol 2, but since there are more typically developing participants in protocol 1 and more SOD− participants in protocol 2 and that the SOD− is the group with the lowest values, it does not explain the SOD− versus TYP difference we observed.

#### Correlations Between the AF Properties and Language/Communication Scores

We investigated whether the AF properties were related to specific verbal abilities by examining the relationship between the AF volume, number of streamlines, or FA and the Verbal IQ (VIQ) measures or ADOS Communication scores separately for each group. We used the Comprehension and Similarities subtests, the 2 Verbal IQ subtests, which gave different scores for the 2 AS subgroups (Table 1). The SOD− subgroup scored higher on all the verbal subtests than the SOD+ subgroup, but the difference was only significant in our sample for the Comprehension scores (*P* = 0.010). As expected, the strongest subtest for the SOD+ group was Block Design. There was no relationship between the cognitive measures and the AF in the TYP group (*P* > 0.329), but smaller AF measures in the AS-SOD− group correlated with lower nonverbal intelligence (PIQ, Block Design subtest, and the Raven) scores only (Pearson’s correlations between AF volume and PIQ: *r* = 0.532, *P* = 0.034; BD: *r* = 0.699, *P* = 0.008; Raven: *r* = 0.687, *P* = 0.005; VIQ: *r* = 0.470, *P* = 0.066; Comp.: *r* = 0.340, *P* = 0.256, Simil.: *r* = 0.385, *P* = 0.194). The AF properties were not related to any intelligence measure in the AS-SOD+ group (*P* > 0.133). However, a higher FA value of the AF correlated with higher severity scores on the ADOS Communications measures (*r* = 0.584, *P* = 0.018).

#### Age Effects

**Figure 4:**
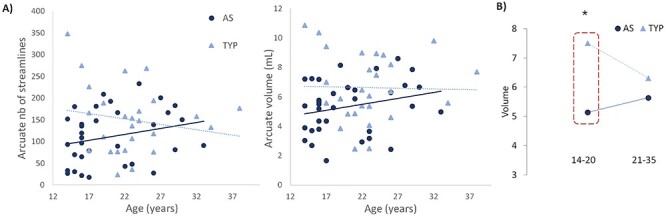
(*A*) Relationship between age and AF size showing group interaction (*B*) Young (14–20 years old): 20 AS, 9 TYP versus older (21–35 years old): 14 AS, 19 TYP. The difference in AF volume is only significant in the younger group and normalizes in older participants.

The relationship between age and AF properties differed between groups (Fig. 4*A*). We investigated the AF properties in the younger and older halves of our sample separately because of the large age range (14–35 years old) of participants included in the study and the fact that brain differences observed in most autism MRI studies appear to normalize into adulthood (Kleinhans et al. 2012) (Fig. 4*B*). The age groups were created by splitting the sample in 2: those < 21 years of age (median age of the sample, *n* = 20 AS, 9 TYP) and those ≥21 (*n* = 14 AS, 19 TYP). The reductions in the AF properties in autism were only observed in the younger group (young AS < young TYP for volume; *t* = −2.462, *P* = 0.031) and nb streamlines (*t* = −2.577, *P* = 0.026); older AS ≈ older TYP for volume (*t* = −1.022, *P* = 0.315) and nb streamlines (*t* = −0.721, *P* = 0.476). Secondary analyses with the AS subgroups (TYP, SOD+, SOD−) including age as a covariate in an ANCOVA increased the significance of the results presented in Effect of SOD Section above (AF volume: *F* = 3.763, *P* = 0.029).

### VBM

There was no significant between-group difference for the whole-brain or ROI analyses. The relationship between the gray matter density of each frontal and temporal ROI and other measures were investigated within each group to help interpret the group-level differences in structural brain connectivity between those regions. In AS-SOD−, there was no association between the white matter properties of the AF and the gray matter density of the cortical areas connected by the AF (see Supplementary Table 3 for correlation values). However, there was a strong positive correlation between the Comprehension and Similarities subtests and total VIQ score and the gray matter density of all language processing areas (frontal and temporal) connected by the AF. In the AS-SOD+ subgroup, the age of first words and sentences positively correlated with the gray matter density of the temporal auditory processing (BA 22). In the same group, the gray matter density of the frontal and temporal ROIs negatively correlated with the Similarities subtest scores (opposite pattern from that of the SOD− group), as well as with the ADOS Social and Communication scores, but was unrelated to VIQ.

## Discussion

### Summary of the Findings

We used diffusion imaging tractography to assess autism/control differences in the AF in adolescent and adult autistic individuals of typical intelligence, with or without SOD. In typical development, the left AF is associated with word acquisition, growth of the vocabulary (Su et al. 2018), and linguistic abilities (Salvan et al. 2017). Accordingly, lesions in the white matter forming the AF in typical adults cause deficits in the repetition of speech and can also impair speech monitoring and learning (Damasio and Damasio 1980; Bernal and Ardila 2009). AS individuals as a group showed reduced anatomical connectivity between the left frontal and temporal language-processing areas of the brain compared with the typically developing group. The reduction of the arcuate volume and number of streamlines was driven by the AS subgroup without SOD (SOD−), whereas the AS-SOD+ subgroup was similar to the typical group in this regard. This difference in connectivity was not related to gray matter density of the frontal or temporal areas connected by the AF and was more pronounced in younger individuals. This is the first study to investigate specifically the anatomical white matter connections between the classical frontal and temporal language areas of the brain in well-characterized AS subgroups and to show that different speech development trajectories in AS may be associated with the establishment of different structural brain networks.

### Accordance With Previous Imaging Studies Comparing Brain Connectivity and Activation Among SOD+ and SOD− Autistic Participants

A recent resting-state functional connectivity study showed atypical brain connectivity between language areas and other brain regions including visual areas in AS individuals between 8 and 18 years old but no intrahemispheric group difference between the frontal and temporal language areas (Gao et al. 2019). The authors also found that lower language abilities were associated not with reduced connectivity within the classical language network but with reduced connectivity between the posterior cingulate cortex and the visual areas, the former acting as a mediator between visual areas and the inferior frontal area in those individuals. In our study, and counterintuitively, AS individuals with the most typical speech development milestones are those that present the largest differences relative to typical populations. Moseley et al. (2016) also found a smaller AF volume in individuals diagnosed with Asperger syndrome (and therefore without SOD, according to DSM-IV criteria) than in nonautistic individuals. In a functional MRI study, Samson et al. (2015) exposed SOD+ and SOD− AS individuals to speech-like sounds. The AS-SOD+ group displayed higher activation in the primary auditory cortex, consistent with hyperprocessing of the acoustical properties of speech, but no differences in higher-level language areas, relative to the typical group. In contrast, the AS-SOD− group showed activation of a greater area of the brain surface centered on language processing regions, including the IFG (Broca’s area) and the middle temporal gyrus, the main areas connected by the fibers of the AF. This was interpreted as functional evidence of cortical rededication, increasing the brain surface dedicated to language function in AS-SOD− individuals, as no anatomical difference in gray matter density or volume could explain this increased activity.

### Relationship Between Structural Subgroup-Level Differences and Intelligence Profiles

The 2 AS subgroups under study differed not only by their speech development history, but also in their respective peaks of abilities in the Wechsler scales of intelligence. The Block Design subtest, in the Performance IQ scale, is a relative strength in AS-SOD+ individuals, whereas the Similarities and Comprehension subtests are relative strengths in the Verbal IQ scale for the SOD− subtype (Soulieres et al. 2011; Nader et al. 2015). Lower AF volumes in AS-SOD− correlated with the nonverbal Performance IQ and Block Design subtests but were not related to the Verbal IQ subtests. This suggests that the anatomical connectivity between the temporal and frontal language areas is not a significant contributor to language development in the SOD− subtype. Better Verbal IQ in this subgroup was associated with higher gray matter density (measured using VBM) in the IFG and temporal areas. Verbal IQ was not associated with gray matter density measures in the SOD+ subgroup. Cerebral reorganization in the SOD− subtype, while preserving speech function, could explain the contrasted pattern among autistic subgroups. Such reorganization would lead to less interdependence of the frontal and temporal language areas and thus less connectivity between them. Localized atypicalities rather than abnormalities in long-range connections could also represent an alternative explanation for the lack of relationship between AF properties and speech onset variations in the AS-SOD+ group (Haigh et al. 2020).

### Effect of Age

A smaller AF in the SOD− autistic subgroup was mostly observed in younger individuals. This suggests that language development in AS is subtended by atypical brain development processes, even when resulting in a normal outcome. This is consistent with the notion of an alternative neurodevelopmental trajectory for brain connectivity in AS (Muller et al. 2011; Kleinhans et al. 2012; Uddin et al. 2013; Nomi and Uddin 2015; Picci et al. 2016). For example, Nomi and Uddin (2015) reported hyperconnectivity within brain networks but hypoconnectivity between networks in AS children under 11 years of age, but an opposite pattern in autistic adolescents and no difference in adults. A generalization from one age group or from a large age range group may therefore result in contradictory or uninformative conclusions. Longitudinal studies or those investigating age groups separately are needed to clarify the relationship between speech acquisition history and the functions of the language processing areas of the brain and how development differentially affects their connectivity in all AS subgroups.

### How a Reduced AF Fits With Underconnectivity Theories of Autistic Brain Development

The early underconnectivity theories of autism were grounded on a distance rule, stipulating that the posterior and anterior brain areas were underconnected, whereas more local networks were overconnected. More recent evidence and literature reviews now agree that over- and underconnectivity are both present in the autistic brain and discrepancies in results greatly depend on methodological aspects (e.g., intrinsic vs. task functional magnetic resonance imaging [fMRI] connectivity, whole-brain vs. ROI analyses), diagnostic heterogeneity, and the age of the samples (Muller et al. 2011; Picci et al. 2016). The direction of connectivity differences also depends on the targeted anatomical and/or functional areas (e.g., primary sensory vs. higher-order processing areas) (Kana et al. 2014; Keown et al. 2017). In autism, the hierarchy between perceptual and higher-order networks is modified and possibly idiosyncratically organized: atypical brain growth would affect how the functional networks are spatially arranged (Keown et al. 2017; Hong et al. 2019a). This apparent noise becomes much clearer when separating the individuals according to a proxy of their speech history (Hong et al. 2019b).

### The SOD+/SOD− Difference: Similar Enhanced Plasticity, but Different Targets?

As all the autistic participants of the current study developed proficient speech and were verbal at the time of testing, our results suggest that AS-SOD− individuals may use alternative networks or cortical allocation for developing speech early, associated with a less developed AF. In contrast, AS-SOD+ individuals may develop their speech in association with the AF but later in development. AS-SOD+ individuals display good visuospatial skills and perceptual peaks of abilities, including the Block Design subtest of the Wechsler (Caron et al. 2006), achieved with less reliance on frontal processes or in a more independent fashion (see Samson et al. 2012 for a meta-analysis). Such enhanced perceptual functioning in the visual modality is observed in the form of increased occipital activation, even in higher-order cognitive tasks that are not purely perceptual (Soulieres et al. 2009). Such atypical brain connectivity may result in (or be caused by) more independent or autonomous functioning of the perceptual brain areas (Just et al. 2012; Kana et al. 2013). According to a prior model of autism (Mottron et al. 2014), genetic differences associated with brain plasticity target the most evolutionarily variable brain regions, the multimodal association cortices (Mueller et al. 2013). In AS-SOD−, the language areas are most affected by such plastic reorganization, whereas the associative perceptual areas are targeted in AS-SOD+. The reduced connectivity observed here between the language processing regions for the SOD− subgroup, together with the finding of enhanced brain activation in the language processing areas (Samson et al. 2015), parallels the functional and structural pattern reported in the visual modality in the SOD+ subtype. The superior performance in visuospatial tasks and activation of visual expertise regions in the SOD+ subtype and the language peaks and superior activation/limited AF connectivity in the AS-SOD− subgroup converge toward an overarching model in which functional regions related to the domain of expertise may function more autonomously in autism.

### Limitations

The cross-sectional nature of this study could not determine whether our observation of a larger AF in SOD+ compared with SOD− AS is experience-dependent or naturally bigger from the beginning. Although with the wide age range of the participants included in this study, our results allowed to suggest structural age-related differences, further longitudinal studies would help understand how the AF develops in both neurotypical and AS populations. Other limitations include the use of 2 different scanning protocols that can affect output metrics from tractography. This study was intended to better define how having SODs in AS relate to the anatomical connectivity within the language network, which was achieved by having subgroups characterized according to whether they had or not delays in the age of their first words or first sentences, minimizing other confounding variable as much as possible. Consequently, our results cannot be generalized to the whole AS populations and more studies using different inclusion criteria are necessary, for example, to clarify whether the results of the current study also apply to left-handed, females, or lower-functioning individuals.

## Supplementary Material

Supplementary_material_V2_tgaa077

## Notes

We would like to thank all the participants and their families.
