# Supplementary material for: Age of Speech Onset in Autism Relates to Structural Connectivity in the Language Network
Source: Cereb Cortex Commun. 2020 Oct 23;1(1):tgaa077. doi: 10.1093/texcom/tgaa077 (PMC8152885; doi:10.1093/texcom/tgaa077)
Supplement: Supplementary_material_V2_tgaa077 [file supplementary_material_v2_tgaa077.docx]

**Supplementary Material**

|  | **Left Arcuate** | **Protocol 1** | **Protocol 2** | ***t*** | **p** |
| --- | --- | --- | --- | --- | --- |
| **TYP** | Nb (*SD*) | 146.65 (79.0) | 182.50 (93.3) | -1.031 | **.312** |
|  | Vol (*SD*) | 6.40 (2.1) | 7.62 (2.8) | -1.256 | **.220** |
|  | FA (*SD*) | 0.498 (0.03) | 0.506 (0.01) | -0.744 | .464 |
| **AS** | Nb (*SD*) | 102.38 (65.0) | 121.00 (60.9) | -0.858 | .397 |
|  | Vol (*SD*) | 4.98 (1.68) | 5.75 (1.91) | -1.251 | .220 |
|  | FA (*SD*) | 0.495 (0.03) | 0.494 (0.03) | 0.002 | .998 |
| **SOD+** | Nb (*SD*) | 116.00 (67.3) | 129.20 (34.1) | -0.413 | .685 |
|  | Vol (*SD*) | 5.48 (1.6) | 6.63 (0.7) | -1.497 | .154 |
|  | FA (*SD*) | 0.491 (0.3) | 0.507 (0.4) | -0.927 | .368 |
| **SOD-** | Nb (*SD*) | 67.00 | 117.27 | -1.432 | .174 |
|  | Vol (*SD*) | 3.68 | 5.36 | -1.607 | .130 |
|  | FA (*SD*) | 0.50 | 0.48 | 0.769 | .454 |

**Table S1**. Independent sample t-tests comparing MR protocols in each group and subgroup separately.

|  |  | **TYP** | **AS** | ***t*** | **p** |
| --- | --- | --- | --- | --- | --- |
| **Left AF** | nb *(SD)* | 156.89 (83.2) | 111.15 (62.8) | -2.456 | **.017** |
|  | Vol *(SD)* | 6.75 (2.3) | 5.34 (1.8) | -2.662 | **.010** |
|  | FA *(SD)* | 0.500 (0.03) | 0.494 (0.03) | -0.880 | .382 |
| **Right AF** | nb *(SD)* | 102.43 (81.9) | 88.39 (71.0) | -0.621 | .538 |
|  | Vol *(SD)* | 4.78 (2.6) | 4.53 (2.3) | -0.346 | .731 |
|  | FA *(SD)* | 0.518 (0.02) | 0.504 (0.03) | -1.621 | .110 |
| **Left SLF III** | nb *(SD)* | 176.8 (109.12) | 164.9 (89.5) | -0.459 | .648 |
|  | Vol *(SD)* | 6.13 (2.4) | 5.77 (2.1) | -0.621 | .537 |
|  | FA *(SD)* | 0.48 (0.02) | 0.48 (0.02) | -0.742 | .461 |

**Table S2.** Average values of number of streamlines (nb), volume (Vol in mL), and fractional anisotropy (FA) and standard deviations (SD) for each group (Typical (TYP), Autism Spectrum (AS)) and each tract: left and right arcuate fasciculus (AF), left superior longitudinal fasciculus, 3^rd^ branch (SLF III).

|  |  | **SOD-** | **SOD** | **TYP** |
| --- | --- | --- | --- | --- |
| **VIQ** | *BA 44* | ***r*=.609, *p*=.012** | *r*=-.151, *p*=.550 | *r*=.189, *p*=.345 |
|  | *BA 45* | ***r*=.723, *p*=.002** | *r*=-.072, *p*=.777 | *r*=.201, *p*=.314 |
|  | *BA 41-42* | ***r*=.692, *p*=.003** | *r*=-.250, *p*=.316 | *r*=.263, *p*=.184 |
|  | *BA 22* | ***r*=.716, *p*=.002** | *r*=-.284, *p*=.253 | *r*=.203, *p*=.309 |
| **Comprehension** | *BA 44* | ***r*=.783, *p*=.002** | *r*=.008, *p*=.976 | *r*=.248, *p*=.232 |
|  | *BA 45* | ***r*=.797, *p*=.001** | *r*=.001, *p*=.996 | *r*=.279, *p*=.176 |
|  | *BA 41-42* | ***r*=.828, *p*<.001** | *r*=.139, *p*=.583 | *r*=.365, *p*=.073 |
|  | *BA 22* | ***r*=.825, *p*=.001** | *r*=.083, *p*=.742 | *r*=.340, *p*=.096 |
| **Similarities** | *BA 44* | ***r*=.665, *p*=.013** | ***r*=-.527, *p*=.024** | *r*=.125, *p*=.553 |
|  | *BA 45* | ***r*=.682, *p*=.010** | *r*=-.426, *p*=.078 | *r*=.118, *p*=.575 |
|  | *BA 41-42* | ***r*=.723, *p*=.005** | ***r*=-.616, *p*=.007** | *r*=.100, *p*=.634 |
|  | *BA 22* | ***r*=.699, *p*=.008** | ***r*=-.557, *p*=.016** | *r*=.120, *p*=.568 |
| **ADOS Com** | *BA 44* | *r*=.044, *p*=.904 | ***r*=-.769, *p*<.001** | _ |
|  | *BA 45* | *r*=.010, *p*=.979 | ***r*=-.793, *p*<.001** | _ |
|  | *BA 41-42* | *r*=-.121, *p*=.740 | ***r*=-.694, *p*=.003** | _ |
|  | *BA 22* | *r*=.034, *p*=.925 | ***r*=-.680, *p*=.004** | _ |
| **Age of 1st words** | *BA 44* | *r*=.018, *p*=.950 | *r*=.352, *p*=.166 | _ |
|  | *BA 45* | *r*=.001, *p*=.997 | *r*=.348, *p*=.171 | _ |
|  | *BA 41-42* | *r*=-.268, *p*=.353 | *r*=.335, *p*=.188 | _ |
|  | *BA 22* | *r*=-.174, *p*=.552 | ***r*=.598, *p*=.011** | _ |
| **Age of 1st Sent** | *BA 44* | *r*=-.267, *p*=.402 | *r*=.373, *p*=.155 | _ |
|  | *BA 45* | *r*=-.451, *p*=.141 | *r*=.321, *p*=.225 | _ |
|  | *BA 41-42* | ***r*=-.649, *p*=.023** | *r*=.394, *p*=.131 | _ |
|  | *BA 22* | *r*=-.563, *p*=.057 | ***r*=.588, *p*=.017** | _ |

**Table S3.** Correlations for each group between language and communication behavioral measures and grey-matter density values for the frontal (Brodmann areas 44 and 45) and temporal (Brodmann areas 41-42 and 22) language areas. *P*earson’s r and *p* values are presented.
